# *Prevotella histicola* Transplantation Ameliorates Cognitive Impairment and Decreases Oxidative Stress in Vascular Dementia Rats

**DOI:** 10.3390/brainsci13081136

**Published:** 2023-07-29

**Authors:** Rui Duan, Jiankang Hou, Xixi Wang, Zhihang Huang, Haiming Cao, Junya Hu, Qiang Peng, Huijie Duan, Qingguang Wang, Xiangliang Chen

**Affiliations:** 1 Department of Neurology, Nanjing First Hospital, Nanjing Medical University, Nanjing 210006, China; duanruicpu@163.com (R.D.); 15850667704@163.com (J.H.); w15895820585@163.com (X.W.); m13813591402@163.com (Z.H.); cao_haiming@126.com (H.C.); pengqiangkite@163.com (Q.P.); 2 Department of Pharmacy, Nanjing First Hospital, China Pharmaceutical University, Nanjing 210006, China; hujunya0513@163.com (J.H.); dhjsdcpu@163.com (H.D.); 3 Department of Neurology, Jiangyin Hospital Affiliated to Nantong University, Wuxi 214400, China

**Keywords:** *Prevotella histicola*, vascular dementia, inflammation

## Abstract

Vascular dementia is a type of dementia from brain damage caused by cerebrovascular lesions and vascular risk factors. *Prevotella histicola* is a species of *Prevotella*, belonging to the category of obligate anaerobe. The purpose of our work was to study the protection of *Prevotella histicola* on cognitive function in rats subjected to vascular dementia (VaD) and investigate underlying molecular mechanisms. The rats were randomly divided into three groups: control group, 2VO group and 2VO + *Prevotella histicola* group. The VaD rats (the 2VO group and 2VO + *Prevotella histicola* group) were generated by bilateral common carotid artery occlusion (2VO). Rats in the 2VO+ *Prevotella histicola* group were administered with *Prevotella histicola* twice daily. In comparison with the rats in the 2VO group, rats in the 2VO + *Prevotella histicola* group presented an enhanced cognitive ability, increased synapse-associated protein expression, a downregulation of proinflammatory factors and an upregulation of neurotrophic factors. The relevant mechanism of the protective effect of *Prevotella histicola* may be associated with the inhibition of glial cell-associated inflammation by regulating phosphorylation of CaMKII. In conclusion, *Prevotella histicola* attenuates neurological impairments via regulating synapse-associated protein expression and the liberation of inflammatory elements in vascular dementia rats. The findings above might benefit the development of *Prevotella histicola* transplantation as a promising treatment of VaD.

## 1. Introduction

Vascular dementia (VaD) generally means vascular cognitive impairment (VCI), which is a type of dementia from brain damage caused by cerebrovascular lesions and vascular risk factors, and is presently considered the second most prevalent type of dementia, followed by Alzheimer’s disease (AD) [1,2]. Recently, various pharmaceutical preparations approved to treat AD, including donepezil, galantamine and memantine, have proven to be beneficial for cognitive functionality in VaD patients. Meanwhile, non-pharmacological therapies such as exercise and diet are also being studied for VaD patients [3,4]. Nonetheless, there is still no treatment for VaD that is specific and effective.

Gut microbiota is a group of microbes that live in the body’s gut. In general, the homeostasis of the intestinal microbiome is not only involved in maintaining intestinal barrier integrity, but also in regulating the establishment of the body’s metabolism and immune system [5,6,7]. Altered or ecologically disequilibrated gut microbiota is linked to a wide range of central nervous system disorders. It is reported that abnormal alterations in gut microbiota and systemic immune abnormalities promote the development of AD, so the recovery of gut microbiota homeostasis potentially has a positive impact on AD treatment [8]. Additionally, it has been proven that the transplantation of fecal microbiota in Parkinson’s mice attenuates neurological impairment by inhibiting microbiota–intestinal–brain axis-mediated inflammation through the TLR4 signaling pathway [9]. *Prevotella*, one of the prevalent types of the intestinal flora, belongs to the category of obligate anaerobe [10,11,12]. *Prevotella histicola* significantly reduces depression-like behaviors and neuronal damage induced by estrogen deficiency [13]. However, whether *Prevotella histicola* improves vascular dementia-associated neurological behavior impairment remains uncertain.

Neuroinflammation is a glial cell-mediated immune cascade response of the central nervous system. Inflammatory response is accompanied by the pathological process of vascular dementia, and the excessive activation of neuroinflammation will lead to pathological events such as blood–brain barrier injury and neuronal apoptosis, which can provoke or exacerbate the occurrence and progression of VaD [14]. Significantly, it is reported that short-chain fatty acids derived from gut microbiota alleviated post-stroke neurological deficits and inflammation [15]. And, it is verified that gut microbiota remodeling by *B. infantis* reduces brain inflammation and improves neuroplasticity [16]. In addition, *Prevotella histicola* has reported to be a beneficial bacterium that produces short-chain fatty acids and is involved in alleviating disease progression in arthritis [17]. However, whether *Prevotella histicola* transplantation regulates the neuroinflammation to alleviate VaD is yet to be discovered.

Here, in the present study, we established the rat VaD model to assess the beneficial role of *Prevotella histicola* transplantation on VaD and to explore potential mechanisms, and have found that the dysbiosis of gut bacteria is closely related to the pathogenesis of VaD, providing a new perspective on VaD treatment.

## 2. Materials and Methods

### 2.1. Animals and Administration

Male Wistar rats of SPF level, were purchased from Animal Model Center of Nanjing University. Animal experiments were conducted in accordance with the institutional guidelines of NIH Guide for the Care and Use of Laboratory Animals. Moreover, all animal investigations were carried out with the approval of the Ethics Committee of Nanjing First Hospital (license number: DWSY-2104285). 

Eight-week-old rats were then divided randomly into three groups: sham group (n = 30), 2VO group (n = 30) and 2VO + *Prevotella histicola* group (n = 30). Each animal from each group was housed individually. *Prevotella histicola* (DSMZ, Braunschweig, Germany) was fully dispersed into sterile water (10^10^ cfu/g). After 24 h of 2VO surgery, rats in 2VO + *Prevotella histicola* group were administered with the suspension of *Prevotella histicola* (10^10^ cfu/g) twice daily for 6 weeks; meanwhile, rats in the sham group and 2VO group were administered saline in equal volumes at the same frequency. At the sixth week, the behavioral test (Morris water maze) was carried out. *Prevotella histicola* was delivered one time daily, following behavioral assessment. Finally, the rats were euthanized and their hippocampi were separated for subsequent experimental analysis.

### 2.2. Establishment of Vascular Dementia (VaD) Model

After a 12 h fasting/4 h water deprivation, rats were anesthetized and silk threads were used to permanently ligate both common carotid arteries. In the sham-operated group, only bilateral common carotid arteries were separated but not ligated. Rats were fed separately and injected with penicillin (2 × 105 U/day) for 3 consecutive days to avoid postoperative infection. 

### 2.3. Morris Water Maze

Morris water maze (Zhongshidichuang Co., Ltd., Beijing, China) was used in this study. The test was divided into two sections. ① Hidden platform trial: If the rat reached the platform within 60 s, the rat was allowed to remain on it for 30 s and then the next training began. If the rat could not find the platform, the rat was placed on it and allowed to remain there for 30 s before the next training, and a 60 s latency was recorded. Rats were trained 4 times per day for a total of 5 days. Each trial started with a different platform location. When testing was completed, the hair of the rats were dried and the rats were normalized before returning to the animal facility. ② Probe trial: Only one trial with no platform was conducted, and the rats were placed in the quadrant opposite to the hidden platform test to start the trial. The trail length was set at 60 s and the period of time spent by the rat in the platform quadrant was recorded. 

### 2.4. Nissl Staining

Nissl staining was applied to assess the damage to hippocampal neurons. Briefly, paraffin sections were deparaffinized and then rehydrated and later stained with tar violet for 1 h at 56 °C. Afterward, the dye was washed off and dehydrated by immersion in ethanol at various gradients of concentration, and the sections were permeabilized with xylene and sealed with neutral gum. Finally, randomly selected hippocampal areas were photographed with an Olympus microscope (Olympus™, Tokyo, Japan) and then quantitatively analyzed. Nuclei with round cell bodies, cytoplasmic Nissl material and visible nucleoli were identified as normal neurons, whereas nuclei with a disorganized arrangement, Nissl material disappearance, surrounded by voids and unrecognizable nucleoli were identified as damaged neurons. Nissl-positive neurons % = positive neurons/total neurons × 100%.

### 2.5. Oxidative Stress Parameters Measurement

Samples from rat hippocampi were homogenized in cold saline, centrifuged at 4 °C and the supernatant was aspirated to measure the concentrations of glutathione peroxidase (GPX; cat. no. A005-1-1), superoxide dismutase (SOD; cat. no. A001-3-2), protein carbonyl compounds (PCC; cat. no. A087-1-2) and malondialdehyde (MDA; cat. no. A003-1-2) in accordance with the kit protocol. All kits were purchased from the Nanjing Jiancheng Institute of Bioengineering (Nanjing, China).

### 2.6. ELISA 

Rats were sacrificed to isolate the hippocampus tissue. Hippocampus tissues were fully homogenized at 4 °C and the supernatant was aspirated upon centrifugation for 20 min at 3000 rpm/min. The contents of interleukin 1 beta (IL-1β), tumor necrosis factor alpha (TNF-α) and interleukin 1 beta (IL-6) in the hippocampus homogenate supernatant were determined via relevant ELISA kits as per the manufacturer’s instructions. The abovementioned ELISA kits were as follows: rat IL-1 beta DuoSet ELISA (DY501), rat TNF-alpha DuoSet ELISA (DY510) and IL-6 ELISA Kit (R6000B), provided by R&D Systems (Emeryville, CA, USA). 

### 2.7. Western Blot

Hippocampus tissues of rats were homogenized in RIPA buffer and total proteins were extracted by centrifugation at 4 °C and 12, 000 g for 5 min, the concentrations of which were then measured via BCA kit (Thermo Fisher Scientific, Waltham, MA, USA). Following the application of 5 × loading buffer, proteins were degenerated in boiled water, separated by electrophoresis and then transferred to the PVDF membrane. The protein bands were blocked by 5% non-fat dried milk at room temperature for 2 h. Primary rabbit antibodies anti- microtubule-associated protein 2 (MAP-2) (1:1000, #4542; Cell Signaling Technology, Boston, MA, USA), anti-synaptophysin (SYP) (1:1500, #SAB4502906; Sigma-Aldrich, St. Louis, MO, USA), anti-postsynaptic density protein 95 (PSD-95) (1:2000, cat.no.20665-1-AP; Proteintech, Chicago, IL, USA), anti-B cell lymphoma 2 protein (Bcl-2) (1:1000, ab196495; Abcam), anti-BCL2 associated X (Bax) (1:1000, #2772; Cell Signaling Technology), anti-cleaved-caspase-3 (1:1000, #9661; Cell Signaling Technology), calcium/calmodulin-dependent protein kinase II (CaMKII) (1:1000, #4436; Cell Signaling Technology), phospho-CaMKII (1:1000, #12716; Cell Signaling Technology), anti-glial fibrillary acidic protein (GFAP) antibody (1:1000, #45946; Cell Signaling Technology), anti-ionized calcium-binding adaptor molecule 1 (Iba1) antibody (1:1000, #17198; Cell Signaling Technology) and β-actin (1:1000; ab8227; Abcam, Cambridge, UK) were further incubated with the membrane at 4 °C overnight, after which, second antibodies corresponding with HRP and labeled goat-anti-Rabbit IgG (1:5000; Cell Signaling Technology) were introduced. Protein bands were visualized using an ECL kit (Thermo Fisher Scientific, Waltham, MA, USA) and then quantified via normalization to the β-actin level by Image Pro Plus 6.0 software. 

### 2.8. RT-qPCR

Trizol reagents (Invitrogen, Thermo Fisher Scientific) extracted total RNA from the hippocampus tissues of rats. RNA samples were reversely transcribed into cDNA using kits (Takara, Kusatsu, Japan). According to the standard SYBR-Green method, the ABI7500-type sequence detection system (7500, ABI, Austin, TX, USA) was utilized for the assay. β-actin serves as the internal control of mRNA. Primers for PCR comprise β-actin forward (5′-CGGTCAGGTCATCACTATCG-3′) and β-actin reverse (5′-TTCCATACCCAGGAAGGAAG-3′); TNF-α forward (5′-CCACGTCGTAGCAAACCACCAAG-3′) and TNF-α reverse (5′-CAGGTACATGGGCTCATACC-3′); IL-1β forward (5′-CACCTCTCAAGCAGAGCACAG-3′) and IL-1β reverse (5′-GGGTTGCATGGTGAAGTCAAC-3′); IL-6 forward (5′-CCAGTTGCCTTCTTGGGACT-3′) and IL-6 reverse (5′-CTGGTCTGTTGTGGGTGGTA-3′).

### 2.9. Statistical Analysis

Statistical analysis of the experimental data was performed with GraphPad Prism 8.0 (GraphPad Software, San Diego, CA, USA) software. All data were shown as mean ± standard deviation (SD). Differences between groups greater than or equal to three were analyzed using one-way ANOVA and Tukey’s post hoc test. Two-way repeated measures ANOVA and Bonferroni’s multiple comparison test were used to compare the MWM results. Statistical significance was considered when *p* < 0.05.

## 3. Results

### 3.1. Prevotella Histicola Transplantation Improves Neurological Behavior of VaD Model Rats

**Figure 1:**
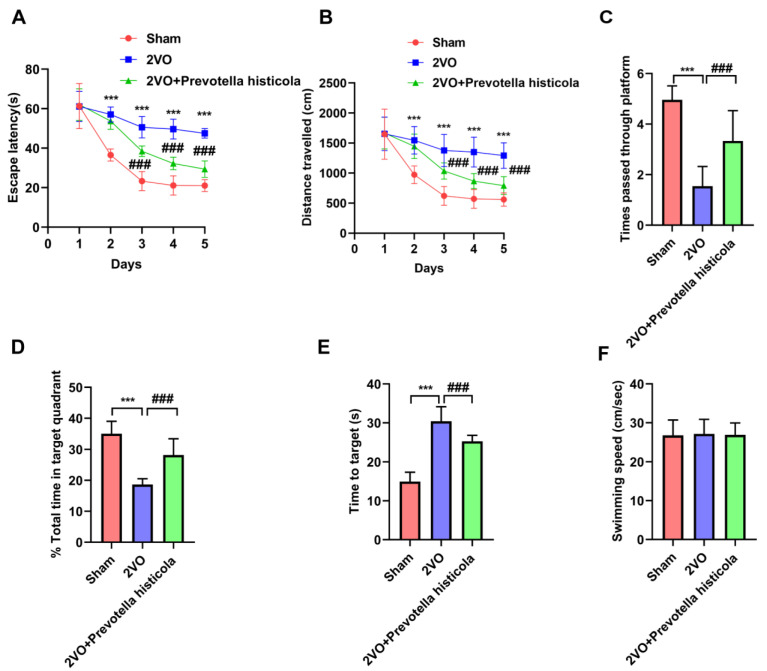
*Prevotella histicola* transplantation improves neurological behavior of VaD model rats. (**A**) The latency, (**B**) exploration distance, (**C**) times passed through platform, (**D**) time in target quadrant, (**E**) time to target and (**F**) swimming speed of rats in different groups (n = 30 per group). Results are shown as mean ± SD. *** *p* < 0.001 versus the sham group; ### *p* < 0.001 versus the 2VO group.

Morris water maze experiment results indicated that compared with the sham-operated group, the escape tendency and distance travelled in the VaD model group (2VO) were all increased, indicating that VaD model rats had developed cognitive dysfunction, while the escape tendency and distance travelled of the 2VO + *Prevotella histicola* group were significantly shortened compared with the 2VO group (Figure 1A,B). Notably, *Prevotella histicola* transplantation significantly reversed the negative effects of 2VO in reducing times passed through platform (Figure 1C) and total time period in target quadrant (Figure 1D). Moreover, the time to target in the 2VO + *Prevotella histicola* group was also shortened, compared with the 2VO group (Figure 1E). But, the swimming speed of rats did not differ between the groups (Figure 1F). These results indicated that *Prevotella histicola* transplantation improved the rat neurological behavior. 

### 3.2. Prevotella Histicola Transplantation Affects Hippocampal Pathological Morphology and Synapse-Associated Protein Expression in VaD Model Rats

**Figure 2:**
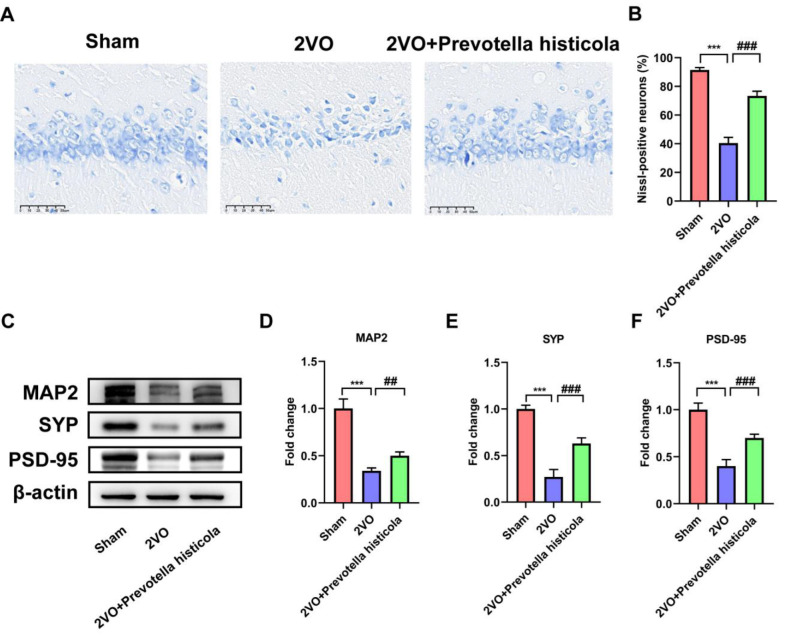
*Prevotella histicola* transplantation affects hippocampal pathological morphology and synapse-associated protein expression in VaD model rats. (**A**) Images reflecting neurons in the hippocampus tissue of the rats across the groups were detected via Nissl staining, bar = 50 μm (n = 6 per group). (**B**) Quantification of the Nissl-positive cells are shown as a bar chart (n = 6 per group). The expression of MAP2 (**C**,**D**), SYP (**C**,**E**) and PSD-95 (**C**,**F**) in hippocampus tissue from the indicated group were detected by Western blot assay; β-actin served as a loading control (n = 6 per group). Results are shown as mean ± SD. *** *p* < 0.001 versus the sham group; ## *p* < 0.01, ### *p* < 0.001 versus the 2VO group.

In Nissl staining, the data displayed that compared with the sham-operated group, the number of hippocampal neurons in the 2VO group decreased, cellular atrophy vacuoles increased and the Nissl-positive neurons decreased significantly. Compared with the 2VO group, the number of hippocampal neurons after *Prevotella histicola* transplantation was increased, the nuclei were enlarged and more Nissl-positive neurons could be seen (Figure 2A,B). Next, we further explored the expression of synapse-associated proteins in VaD model rats. In comparison with the sham group, the levels of MAP2, SYP and PSD-95 were all remarkably downregulated in the 2VO group (Figure 2C–F). Notably, the changes in protein expression induced by VaD were significantly reversed by *Prevotella histicola* transplantation, which indicated that the *Prevotella histicola* transplantation might enhance the synaptic plasticity and ameliorate VaD-induced synaptic damage.

### 3.3. Prevotella Histicola Transplantation Inhibits the Neuronal Apoptosis of Hippocampi in VaD Model Rats

**Figure 3:**
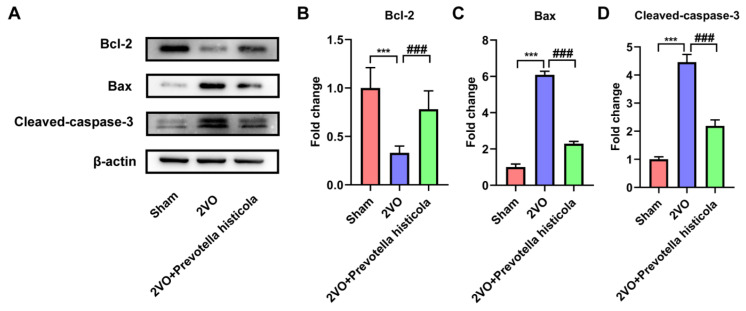
*Prevotella histicola* transplantation inhibits the apoptosis of hippocampal neurons in VaD model rats. (**A**) Representative bands of Bcl-2, Bax and cleaved caspase-3 protein expression within the hippocampi of the rats across the groups. β-actin served as a loading control (n = 6). Quantification for (**B**) Bcl-2, (**C**) Bax and (**D**) cleaved caspase-3 protein expression is shown as a bar chart (n = 6). Results are shown as mean ± SD. *** *p* < 0.001 versus the sham group; ### *p* < 0.001 versus the 2VO group.

We further assessed whether *Prevotella histicola* transplantation inhibited the apoptosis of hippocampal neurons. Therefore, the aim of this research was to investigate the expression of apoptotic proteins in VaD model rat hippocampal tissue. Western blot assay disclosed that the Bcl-2 expression of anti-apoptotic protein was downregulated and the Bax expression of pro-apoptotic protein was upregulated in the 2VO group, and cleaved casepase-3 was upregulated. In contrast, Bcl-2 expression was upregulated after *Prevotella histicola* transplantation, but Bax and cleaved casepase-3 expression were decreased. (Figure 3A–D). The above results proved that *Prevotella histicola* transplantation suppressed hippocampal neuronal apoptosis in VaD model rats.

### 3.4. Prevotella Histicola Transplantation Alleviates Oxidative Stress in Hippocampi of VaD Model Rats

**Figure 4:**
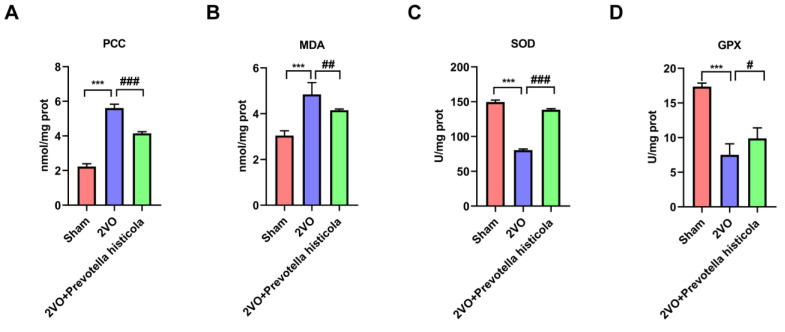
*Prevotella histicola* transplantation alleviates oxidative stress in hippocampi of VaD model rats. (**A**) PCC, (**B**) MDA levels, (**C**) SOD and (**D**) GPX activity in the hippocampi of the rats were measured based on the kit manufacturer’s instructions (n = 6 per group). Results are shown as mean ± SD. *** *p* < 0.001 versus the sham group; # *p* < 0.05, ## *p* < 0.01, ### *p* < 0.001 versus the 2VO group.

Oxidative stress is a key factor in determining neuronal cell damage and loss. To further study the neuro-protective mechanism of VaD rats, we detected the oxidative stress via biochemical detection. The PCC level in the hippocampus was dramatically increased in VaD model rats compared with the sham-operated group, while *Prevotella histicola* transplantation relatively reduced the PCC level (Figure 4A). Compared with the sham-operated group, the biochemical analysis showed that the MDA level was considerably increased in the 2VO group, while *Prevotella histicola* transplantation significantly reduced the levels of MDA (Figure 4B). Instead, the levels of two typical antioxidant enzymes, SOD and GPX, in the hippocampus was substantially decreased in the 2VO group, while *Prevotella histicola* transplantation further strikingly increased SOD and GPX levels (Figure 4C,D). These findings imply that *Prevotella histicola* transplantation could alleviate the oxidative stress damage in the hippocampal neurons of VaD model rats.

### 3.5. Prevotella Histicola Transplantation Regulated the Inflammatory Cytokines after VaD

**Figure 5:**
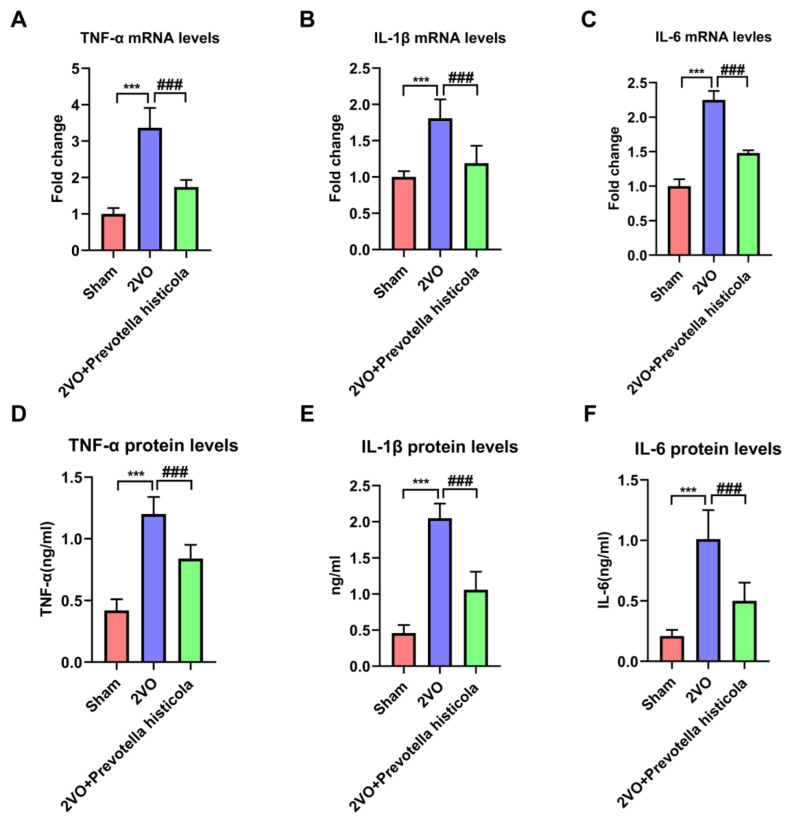
*Prevotella histicola* transplantation regulated the inflammatory cytokines after VaD. The mRNA levels of TNF-α (**A**), IL-1β (**B**) and IL-6 (**C**) in hippocampus tissue was detected by RT-qPCR (n = 6 per group). The protein levels of TNF-α (**D**), IL-1β (**E**) and IL-6 (**F**) in hippocampus tissue was detected by ELISA assay (n = 6 per group). Results are shown as mean ± SD. *** *p* < 0.001 versus the sham group; ### *p* < 0.001 versus the 2VO group.

Firstly, the results of RT-qPCR showed that the mRNA expression of IL-1β, TNF-α and IL-6 were raised greatly in the 2VO group, whose cytokine imbalance was reversed by *Prevotella histicola* in the hippocampi of VaD model rats (Figure 5A–C). Furthermore, the ELISA assay further confirmed that the regulation of these cytokine protein levels in hippocampus tissue by *Prevotella histicola* transplantation was consistent with the above results (Figure 5D–F). In conclusion, *Prevotella histicola* transplantation regulated the inflammatory cytokines after VaD.

### 3.6. Prevotella Histicola Transplantation Regulates the Activation of Microglia and Astrocytes and Affects CaMKII Phosphorylation in the Hippocampi of VaD Rats 

**Figure 6:**
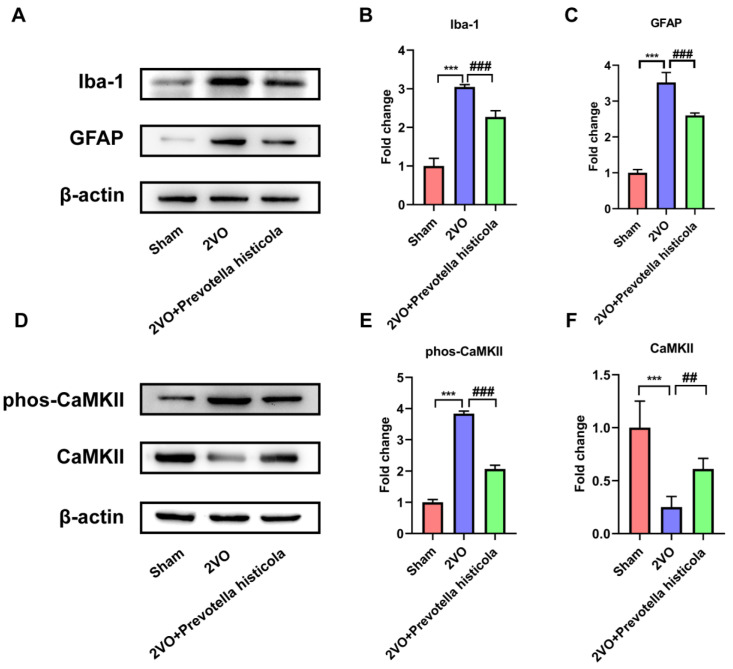
*Prevotella histicola* transplantation regulates the activation of microglia and astrocytes and affects CaMKII phosphorylation in the hippocampi of VaD rats. (**A**) Representative bands of Iba-1 and GFAP protein expression within the hippocampi of the rats across the groups. β-actin served as a loading control (n = 6). Quantification for Iba-1 (**B**) and GFAP (**C**) protein expression is shown as a bar chart (n = 6). (**D**) Representative bands of CaMKII and phospho-CaMKII protein expression within the hippocampi of the rats across the groups. β-actin served as a loading control (n = 6). Quantification for CaMKII (**E**) and phospho-CaMKII (**F**) protein expression is shown as a bar chart (n = 6). Results are shown as mean ± SD. *** *p* < 0.001 versus the sham group; ## *p* < 0.01, ### *p* < 0.001 versus the 2VO group.

We further explored whether *Prevotella histicola* transplantation can affect the activation of glial cells in VaD model rats. Western blot analysis showed significant increases in Iba1, a microglial marker, and GFAP, an astrocyte protein, which were both detected in the 2VO group compared to the sham-operated group of rats, while *Prevotella histicola* transplantation relatively reversed the increased expression of Iba1 and GFAP induced by 2VO (Figure 6A–C). CaMKII is a Ca^2+^-activated enzyme that is highly abundant in the brain, whose phosphorylation is critical for mediating the activation of astrocyte and microglia. We next investigated whether *Prevotella histicola* transplantation was correlated with the phosphorylation of CaMKII. Western blot assay disclosed that compared with the sham-operated group, the level of CaMKII in the 2VO group was downregulated, while the phosphorylation of CaMKII was increased; meanwhile, *Prevotella histicola* transplantation increased CaMKII expression and reversed 2VO-induced CaMKII phosphorylation (Figure 6D–F). The above results suggest that *Prevotella histicola* transplantation regulates hippocampal microglia and astrocyte activation in VaD rats.

## 4. Discussion

Vascular dementia is the dementia stage of vascular cognitive impairment, which can cause serious cognitive impairment [18]. *Prevotella* is a Gram-negative bacterium classified in the genus of symbiotic bacteria [19]. Notably, *Prevotella*-associated imbalances in the body’s gut microbiota influence the pathological mechanisms of a variety of neurodegenerative diseases. It has been certified that reduced levels of butyrate produced by bacteria such as *Prevotella* have been linked to epigenetic changes in neurons in PD patients, as well as the severity of depressive symptoms [20]. Meanwhile, *Prevotella* abundance in WT mice has been found that was appreciably higher than in APP/PS1 transgenic mice of AD [21]. The results in this study confirmed that *Prevotella histicola* transplantation improved the VaD model rats’ neurological behavior (coordinate ability, motor ability, learning and memory ability) comprehensively, which was consistent with the Nissl staining results of the hippocampus in every group.

Oxidative stress-related indicators are one of the biomarkers of VaD [22,23]. Oxidative stress is marked by the overproduction of oxidative mediators and imbalance of antioxidant systems [24,25]. During VaD, the consumption of endogenous antioxidants and the overproduction of ROS increase oxidative damage to proteins, lipids and DNA and further aggravate the course of the disease [26,27,28]. Currently, multiple studies have identified that the inhibition of oxidative stress can improve VaD-induced cognitive dysfunction and neuronal damage. For example, Du et al. verified that the inhibition of TXNIP-associated oxidative stress attenuated cognitive and memory deficits in VaD rats [29]. Meanwhile, it has been reported that Nrf2-mediated antioxidant response reduced the loss of hippocampal neurons to improve cognitive dysfunction in VaD rats [30]. For this study, we examined the PCC, MDA, SOD and GPX levels in the hippocampi of VaD model rats to measure the degree of oxidative stress. We found that VaD initiated oxidative stress activation, generated excess PCC and MDA, while reducing the levels of SOD and GPX. And yet, *Prevotella histicola* transplantation reversed these situations. Meanwhile, we also found that *Prevotella histicola* transplantation inhibits the neuronal apoptosis of hippocampi in VaD model rats. Thus, the inhibition of the activation of oxidative stress during the VaD phase may explain the neuroprotective effect of *Prevotella histicola*.

Inflammatory factor is another important biomarker of VaD [31]. In the early course of VaD, microglia and astrocytes are activated successively, resulting in the release of large amounts of inflammatory cytokines, which trigger a serious inflammatory response and aggravate neuronal damage [32]. Additionally, Liu et al. confirmed that the inhibition of microglia and astrocyte activation significantly reduced neurological damage in ischemic stroke [33]. It was reported that microglia activation exacerbates white matter damage through complement C3/C3aR pathway in response to chronic cerebral hypoperfusion [34]. In this work, we investigated the influence of *Prevotella histicola* transplantation in VaD rats on neuroinflammatory induced by the activation of microglia and astrocyte. We identified that *Prevotella histicola* transplantation prominently restrained the microglia and astrocyte activation and markedly declined the expression of proinflammatory cytokines (IL-6, IL-1β and TNF-α) in the post-2VO hippocampal region. 

It should be noted that this study has a limitation. The specific mechanism of how *Prevotella histicola* inhibits oxidative stress has not been clarified, and we will conduct further research in the future.

## 5. Conclusions

Our outcomes proved that *Prevotella histicola* transplantation attenuated 2VO-induced cognitive deficits through increasing synapse-associated protein expression, reducing oxidative stress and apoptosis, coupled with decreasing the release of pro-inflammatory cytokines. Notably, we also found that the neuroprotective effect of *Prevotella histicola* transplantation on VaD rats were related with the phosphorylation of CaMKII in the hippocampus. Collectively, our study indicates that *Prevotella histicola* transplantation has neuroprotective effects on VaD in rats, which supports the investigation of this microbiota as a potential therapeutic target in clinical subjects with VaD.

## Data Availability

All data generated or analyzed during this study are available from the corresponding author upon reasonable request.

## References

[1] Iadecola C., Duering M., Hachinski V., Joutel A., Pendlebury S.T., Schneider J.A., Dichgans M. (2019). Vascular Cognitive Impairment and Dementia: JACC Scientific Expert Panel. J. Am. Coll. Cardiol..

[2] van der Flier W.M., Skoog I., Schneider J.A., Pantoni L., Mok V., Chen C.L.H., Scheltens P. (2018). Vascular cognitive impairment. Nat. Rev. Dis. Primers.

[3] Farooq M.U., Min J., Goshgarian C., Gorelick P.B. (2017). Pharmacotherapy for Vascular Cognitive Impairment. CNS Drugs.

[4] Zanon Zotin M.C., Sveikata L., Viswanathan A., Yilmaz P. (2021). Cerebral small vessel disease and vascular cognitive impairment: From diagnosis to management. Curr. Opin. Neurol..

[5] Adak A., Khan M.R. (2019). An insight into gut microbiota and its functionalities. Cell. Mol. Life Sci. CMLS.

[6] Schoeler M., Caesar R. (2019). Dietary lipids, gut microbiota and lipid metabolism. Rev. Endocr. Metab. Disord..

[7] Liu X., Chen Y., Zhang S., Dong L. (2021). Gut microbiota-mediated immunomodulation in tumor. J. Exp. Clin. Cancer Res. CR.

[8] Kim M.S., Kim Y., Choi H., Kim W., Park S., Lee D., Kim D.K., Kim H.J., Choi H., Hyun D.W. (2020). Transfer of a healthy microbiota reduces amyloid and tau pathology in an Alzheimer’s disease animal model. Gut.

[9] Zhao Z., Ning J., Bao X.Q., Shang M., Ma J., Li G., Zhang D. (2021). Fecal microbiota transplantation protects rotenone-induced Parkinson’s disease mice via suppressing inflammation mediated by the lipopolysaccharide-TLR4 signaling pathway through the microbiota-gut-brain axis. Microbiome.

[10] Chen C., Fang S., Wei H., He M., Fu H., Xiong X., Zhou Y., Wu J., Gao J., Yang H. (2021). *Prevotella* copri increases fat accumulation in pigs fed with formula diets. Microbiome.

[11] Larsen J.M. (2017). The immune response to *Prevotella* bacteria in chronic inflammatory disease. Immunology.

[12] Li J., Zhao F., Wang Y., Chen J., Tao J., Tian G., Wu S., Liu W., Cui Q., Geng B. (2017). Gut microbiota dysbiosis contributes to the development of hypertension. Microbiome.

[13] Huang F., Liu X., Xu S., Hu S., Wang S., Shi D., Wang K., Wang Z., Lin Q., Li S. (2021). *Prevotella histicola* Mitigated Estrogen Deficiency-Induced Depression via Gut Microbiota-Dependent Modulation of Inflammation in Ovariectomized Mice. Front. Nutr..

[14] Poh L., Sim W.L., Jo D.G., Dinh Q.N., Drummond G.R., Sobey C.G., Chen C.L., Lai M.K.P., Fann D.Y., Arumugam T.V. (2022). The role of inflammasomes in vascular cognitive impairment. Mol. Neurodegener..

[15] Lee J., d’Aigle J., Atadja L., Quaicoe V., Honarpisheh P., Ganesh B.P., Hassan A., Graf J., Petrosino J., Putluri N. (2020). Gut Microbiota-Derived Short-Chain Fatty Acids Promote Poststroke Recovery in Aged Mice. Circ. Res..

[16] Jena P.K., Setayesh T., Sheng L., Di Lucente J., Jin L.W., Wan Y.Y. (2022). Intestinal Microbiota Remodeling Protects Mice from Western Diet-Induced Brain Inflammation and Cognitive Decline. Cells.

[17] Balakrishnan B., Luckey D., Bodhke R., Chen J., Marietta E., Jeraldo P., Murray J., Taneja V. (2021). *Prevotella* histicola Protects From Arthritis by Expansion of Allobaculum and Augmenting Butyrate Production in Humanized Mice. Front. Immunol..

[18] Iadecola C. (2013). The pathobiology of vascular dementia. Neuron.

[19] Sharma G., Garg N., Hasan S., Shirodkar S. (2022). *Prevotella*: An insight into its characteristics and associated virulence factors. Microb. Pathog..

[20] Xie A., Ensink E., Li P., Gordevičius J., Marshall L.L., George S., Pospisilik J.A., Aho V.T.E., Houser M.C., Pereira P.A.B. (2022). Bacterial Butyrate in Parkinson’s Disease Is Linked to Epigenetic Changes and Depressive Symptoms. Mov. Disord. Off. J. Mov. Disord. Soc..

[21] Shen L., Liu L., Ji H.F. (2017). Alzheimer’s Disease Histological and Behavioral Manifestations in Transgenic Mice Correlate with Specific Gut Microbiome State. J. Alzheimer’s Dis..

[22] Raz L., Knoefel J., Bhaskar K. (2016). The neuropathology and cerebrovascular mechanisms of dementia. J. Cereb. Blood Flow Metab. Off. J. Int. Soc. Cereb. Blood Flow Metab..

[23] Barone E., Di Domenico F., Perluigi M., Butterfield D.A. (2021). The interplay among oxidative stress, brain insulin resistance and AMPK dysfunction contribute to neurodegeneration in type 2 diabetes and Alzheimer disease. Free. Radic. Biol. Med..

[24] Dumitrescu L., Popescu-Olaru I., Cozma L., Tulbă D., Hinescu M.E., Ceafalan L.C., Gherghiceanu M., Popescu B.O. (2018). Oxidative Stress and the Microbiota-Gut-Brain Axis. Oxidative Med. Cell. Longev..

[25] Merelli A., Repetto M., Lazarowski A., Auzmendi J. (2021). Hypoxia, Oxidative Stress, and Inflammation: Three Faces of Neurodegenerative Diseases. J. Alzheimer’s Dis..

[26] Cobb C.A., Cole M.P. (2015). Oxidative and nitrative stress in neurodegeneration. Neurobiol. Dis..

[27] Hernández J.A., López-Sánchez R.C., Rendón-Ramírez A. (2016). Lipids and Oxidative Stress Associated with Ethanol-Induced Neurological Damage. Oxidative Med. Cell. Longev..

[28] Yuan T.F., Gu S., Shan C., Marchado S., Arias-Carrión O. (2015). Oxidative Stress and Adult Neurogenesis. Stem Cell Rev. Rep..

[29] Du S.Q., Wang X.R., Zhu W., Ye Y., Yang J.W., Ma S.M., Ji C.S., Liu C.Z. (2018). Acupuncture inhibits TXNIP-associated oxidative stress and inflammation to attenuate cognitive impairment in vascular dementia rats. CNS Neurosci. Ther..

[30] Wang X.R., Shi G.X., Yang J.W., Yan C.Q., Lin L.T., Du S.Q., Zhu W., He T., Zeng X.H., Xu Q. (2015). Acupuncture ameliorates cognitive impairment and hippocampus neuronal loss in experimental vascular dementia through Nrf2-mediated antioxidant response. Free. Radic. Biol. Med..

[31] Custodero C., Ciavarella A., Panza F., Gnocchi D., Lenato G.M., Lee J., Mazzocca A., Sabbà C., Solfrizzi V. (2022). Role of inflammatory markers in the diagnosis of vascular contributions to cognitive impairment and dementia: A systematic review and meta-analysis. GeroScience.

[32] Karve I.P., Taylor J.M., Crack P.J. (2016). The contribution of astrocytes and microglia to traumatic brain injury. Br. J. Pharmacol..

[33] Liu M., Xu Z., Wang L., Zhang L., Liu Y., Cao J., Fu Q., Liu Y., Li H., Lou J. (2020). Cottonseed oil alleviates ischemic stroke injury by inhibiting the inflammatory activation of microglia and astrocyte. J. Neuroinflammation.

[34] Zhang L.Y., Pan J., Mamtilahun M., Zhu Y., Wang L., Venkatesh A., Shi R., Tu X., Jin K., Wang Y. (2020). Microglia exacerbate white matter injury via complement C3/C3aR pathway after hypoperfusion. Theranostics.

